# CpG-ODN Attenuates Pathological Cardiac Hypertrophy and Heart Failure by Activation of PI3Kα-Akt Signaling

**DOI:** 10.1371/journal.pone.0062373

**Published:** 2013-04-30

**Authors:** Liang Yang, Xiangyu Cai, Jie Liu, Zhe Jia, Jinjin Jiao, Jincai Zhang, Changlin Li, Jing Li, Xiang D. Tang

**Affiliations:** 1 Department of Pharmacology, Nankai University School of Medicine, Tianjin, China; 2 Chinese Ministry of Education Key Laboratory of Bioactive Materials, Nankai University School of Medicine, Tianjin, China; University Heart Center Freiburg, Germany

## Abstract

Phosphoinositide-3-kinase α (PI3Kα) represents a potential novel drug target for pathological cardiac hypertrophy (PCH) and heart failure. Oligodeoxynucleotides containing CpG motifs (CpG-ODN) are classic agonists of Toll-like receptor 9 (TLR9), which typically activates PI3K-Akt signaling in immune cells; however, the role of the nucleotide TLR9 agonists in cardiac myocytes is largely unknown. Here we report that CpG-ODN C274 could both attenuate PCH and improve cardiac dysfunction by activating PI3Kα-Akt signaling cascade. *In vitro* studies indicated that C274 could blunt reactivation of fetal cardiac genes and cell enlargement induced by a hypertrophic agent, isoproterenol. The anti-hypertrophic effect of C274 was suppressed by a pan-PI3K inhibitor, LY294002, or a small interfering RNA targeting PI3Kα. *In vivo* studies demonstrated that PCH, as marked by increased heart weight (HW) and cardiac ANF mRNA, was normalized by pre-administration with C274. In addition, Doppler echocardiography detected cardiac ventricular dilation, and contractile dysfunction in isoproterenol-treated animals, consistent with massive replacement fibrosis, reflecting cardiac cell death. As expected, pre-treatment of mice with C274 could prevent cardiac dysfunction associated with diminished cardiac cell death and fibrosis. In conclusion, CpG-ODNs are novel cardioprotective agents possessing antihypertrophic and anti-cell death activity afforded by engagement of the PI3Kα-Akt signaling. CpG-ODNs may have clinical use curbing the progression of PCH and preventing heart failure.

## Introduction

Heart failure remains a leading cause of mortality worldwide despite the broad use of angiotensin-converting enzyme inhibitors (ACEI), β-adrenoceptor blockers, and aldosterone antagonists [1], [2]. Novel preventive and therapeutic strategies are required to better combat this deadly terminal disease status and improve quality of life for the affected.

Heart failure occurs as choreography of pathological cardiac hypertrophy (PCH) and cardiac cell death, with PCH coming into play first. Indeed, PCH is an independent poor predictor of cardiovascular mortality and recognized as a new therapeutic target for heart failure [3], [4]. PCH develops as a result of persistent hypertension, acute myocardial infarction, genetic cardiomyopathy, and diabetes. It is characterized by cell volume increase, metabolic and biochemical abnormality, and reactivation of fetal cardiac genes such as atrial natriuretic factor (ANF) and β-myosin heavy chain (β-MHC) [5], [6]. Thus, because PCH, in essence, is a maladaptive response from the very beginning, it is doomed to heart failure as unmatched cardiac cell death and fibrosis come into play.

Despite complicated mechanisms underlying PCH, a lipid kinase, phosphoinositide 3-kinase γ (PI3Kγ), plays a key role. This class I_B_ PI3K, a heterodimer of p110γ and an adaptor subunit, is activated by Gβγ subunit of G proteins. It is well known that G protein-coupled receptors (GPCRs) are largely responsible for the prohypertrophic effect of major hypertrophic agents including noradrenaline, angiotensin II, and endotheilin-1 [7]. The activated PI3Kγ in turn recruits downstream prohypertrophic mediators such as Akt. Thus, mice with genetic knockout of PI3Kγ are resistant to isoproterenol-induced PCH and heart dysfunction, accompanied by attenuated activation of Akt and extracellular signal-regulated kinase 1/2 (ERK1/2) signaling pathways [8].

Cardiac myocytes also undergo physiological cardiac growth (PCG) as occurred in normal postnatal cardiac growth, physical exercise, or during pregnancy [3], [9], and boosting PCG by exercise is also proposed as a novel means to antagonize PCH and improve impaired cardiac function [10], [11]. Unlike PCH being irreversible, PCG is completely reversible and characterized by enhanced cardiac performance without any obvious cell death and fibrosis [12]. PCG is mediated by class I_A_ PI3Ks, including PI3Kα, PI3Kβ and PI3Kδ, which is not activated by GPCRs but by insulin-like growth factor-1 (IGF-1) or other receptor tyrosine kinases/cytokine receptors [7]. In the heart, PI3Kα is the dominant isoform, which plays a critical role in exercise-induced PCG in addition to antagonizing PCH [13]. It has been reported that transgenic PI3Kα mice were resistant to PCH and cardiac dysfunction induced by pressure overload [14]. Overexpression of PI3Kα in mice with dilated cardiomyopathy also delayed the onset of heart failure, and improve mice lifespan [15]. Thus, activation of the PI3Kα signaling could be a preventive and therapeutic strategy for PCH and heart failure.

Oligodeoxynucleotides containing CpG motifs (CpG-ODN) are synthetic agonists for Toll-like receptor 9 (TLR9), stimulating the innate immune system [16]. Many CpG-ODNs have been developed for the treatment of allergies, cancers, and chronic infections. Recently, TLR9 was reported to reside in cardiac myocytes [17], and CpG-ODNs could induce a strong activation of NFκB and iNOS in cardiomyocytes [18]. In the present study, we have demonstrated that CpG ODN can significantly regress cardiac hypertrophy induced by isoproterenol, in the absence of deleterious effects on fetal gene reactivation and cell size enlargement in vitro. Furthermore, we found that inhibition of PI3K resulted in suppression of the protection effects of CpG ODN. In vivo, it has been found that the injection of CpG ODN could retard the ISO-induced morphological and echocardiographic changes. And there was a significant increase of phosphorylated Akt in the hearts of CpG ODN-treated mice, which indicated that up-regulation of PI3Kα-Akt pathway may contribute to the anti-cardiac hypertrophy activity.

## Materials and Methods

### Animals and cell preparation

Neonatal Sprague-Dawley (SD) rats and C57BL/6 mice were used throughout the experiment and purchased from the Chinese Military Academy of the Medical Science Laboratory Animal Center (Beijing, China). All animals were anesthetized with diethyl ether before each experiment and efforts were made to minimize their suffering. Isolation and culture of neonatal rat ventricular myocytes (NRVM) were conducted using the overnight trypsin-collagenase digestion method as described in our recent publications [6], [19]. The experiments with NRVMs were performed on 2–4 d cultures when synchronously contracting cells were observed. All animal experiments were performed strictly under the guidelines on laboratory animals of Nankai University and the animal protocols were approved by the Institute Research Ethics Committee at the Nankai University (Permit No: 10011).

### CpG-ODNs

Single-stranded oligodeoxynucleotides (ODNs) were synthesized and purified in Takara Company (Daliang, China). The following CpG-ODNs were used: 1585 (5′-ggGGTCAACGTTGAGggggg-3′), 1826 (5′-tccatgacgttcctgacgtt-3′), C274 (5′-tcgtcgaacgttcgagatgat-3′). Lower case and capital letters represent phosphorothioate and phosphodiester linkage, respectively. All CpG-ODNs were diluted in PBS buffer before each use and had no detectable endotoxin as verified using Limulus amebocyte lysate assays (Associates of Cape Cod, Inc).

### Experimental groups and treatment

C57BL/6 mice were randomly divided into 4 groups. (i) NS group (n = 6): normal saline was given i.p. for 6 days; (ii) CpG ODN group (n = 6): C274 (5 mg/kg) was given i.p. for 6 days; (iii) ISO group (n = 8): to induce cardiac hypertrophy, ISO (50 mg/kg) was administered s.c. for 6 days; (iv) CpG ODN/ISO group (n = 8): C274 was injected i.p. for 6 days at a dose of 5 mg/kg/day, 3 h prior to ISO 5 mg/kg/day. After 6 days of treatment, echocardiographic measures of hearts were performed under isoflurane inhalational anaesthesia. Then, under pentobarbitone anaesthesia, animals were sacrificed and hearts were removed, trimmed and washed. The expression of ANF mRNA in the hearts was determined by qPCR. Heart weight (HW) and HW/Body weight (BW) were measured and histological sections of hearts were determined by H-E staining.

### Small RNA interference (siRNA)

siRNA was performed using the standard method as described in our recent publication [6], [20]. All nucleotides were synthesized and 2′ O-methyl modified by GenePharma (Shanghai). For each gene at least two siRNA sequences were designed targeting the coding regions and selected based on silencing efficacy as verified by the reverse transcription-polymerase chain reaction (RT-PCR). For transient transfection cells were incubated for 6 h in transfection medium composed of serum-free DMEM, 2 mM glutamine supplemented with Lipofectamine 2000 (Invitrogen, Shanghai) according to the manufacturer's instructions. Cells were then left in incubation medium containing serum-free DMEM, 2 mM glutamine, 1×ITS Liquid Media Supplement (Sigma-Aldrich), 100 µg/ml penicillin and 100 µg/ml streptomycin for 36 h. This was followed by treatment with vehicle, CpG-ODNs or isoproterenol in a 37°C incubator with 5% CO_2_–95% air.

### RT-PCR and quantitative real-time PCR (qPCR)

Total RNA was isolated from hearts or myocytes using Trizol reagent (Invitrogen, Shanghai) as described [6]. For cDNA synthesis 1.0 µg RNA was used and reactions were carried out using the reverse transcription system (Promega, Shanghai). RT-PCR was performed in a Genemate thermal cycler (Jinge Instr, Hangzhou, China). The following primers were used: ANF: 5′-GGGGGTAGGATTGACAGGAT-3′ and 5′-CTCCAGGAGGGTATTCACCA-3′;β-MHC: 5′-CCTCGCAATATCAAGGGAAA-3′ and 5′-TACAGGTGCATCAGCTCCAG-3′; 18-s rRNA: 5′-ACCGCAGCTAGGAATAATGGA-3′ and 5′-GCCTCAGTT CCGAAAACCA-3′. qPCR assays were performed using SYBR Green Master Mix (Takara Bio, Inc.) in a Bio-Rad IQ5 detection system and the cycle threshold (CT) values were automatically determined in triplicates and averaged. All qPCR sample reactions were normalized to 18-s rRNA expression. A standard curve was run with the dilution series of the amplified fragment allowing the mRNA copy number to be calculated.

### Western blot analysis

NRVM were washed twice with ice-cold phosphate-buffered saline (PBS) and scraped from the culture dish. Cells were lysed and incubated for 30 min in ice-cold RIPA buffer containing 150 mM NaCl, 50 mM Tris (pH 7.5), 0.5% deoxycholic acid, 1% NP-40, 0.1% sodium dodecyl sulphate (SDS), 1 mM Na_3_VO_4_, 10 mM NaF and protease inhibitors. Myocardial tissue was taken from the heart and ground to a powder preparation with liquid nitrogen. About 50 mg of each sample was lysed by RIPA buffer as mentioned above, homogenized for 10 min, and incubated in an ice-bath for 30 min. All the samples were centrifuged at 12,000 g for 15 min at 4°C, and the supernatant was finally collected. Protein concentration was measured using BCA protein assay Kit (Rockford, IL, USA). Equal amounts of protein were resolved in 10% SDS–PAGE and transferred to poly-vinylidene fluoride (PVDF) membrane (Millipore). The membranes were incubated overnight at 4°C with a primary antibody. The antibodies used were anti-Akt and anti-phospho-Akt (Ser473) (Cell Signaling Technology). Peroxidase-conjugated anti-rabbit IgG was used as the secondary antibody. Band densities were quantified using ImageJ.

### Indirect immunofluorescence

Indirect immunofluorescent staining was conducted essentially the same as described [6]. NRVM were grown on laminin-coated glass coverslips and fixed in 4% paraformaldehyde followed by permeabilization with 0.5% Triton X-100. After blocking in 1% BSA-containing PBS, cells were incubated with the primary antibody and subsequently with the secondary antibody (Invitrogen, Shanghai). Images were collected and analyzed on a TCS-SP confocal laser microscopy (Leica, Germany). For surface area determination ImageJ was used and at least 50 individualized cells were analyzed for each experiment.

### Echocardiography

Echocardiography (Visualsonic Vevo 2100™, 30 MHz linear signal transducer) was performed under isoflurane/oxygen anasthesia 24 h after the last drug injection. Averaged M-mode measurements from parasternal long-axis images were recorded. Interventricular septal (IVS), left ventricular posterior wall (LVPW) dimensions, left ventricular internal dimensions (LVID) were taken in diastole and systole. Fractional shortening (FS) was calculated as (LVIDd- LVIDs)/LVIDd×100 and ejection fraction (EF) as (LVIDd^3^- LVIDs^3^)/LVIDd^3^×100.

### Statistical analysis

Results are expressed as means ± SD. One-way ANOVA followed by LSD test or Student's *t* test was performed as implemented in IgorPro (Wavemetrics, Oregon) [21]. A value of *p*<0.05 was accepted as statistically significant.

## Results

### 1. CpG-ODNs inhibit fetal cardiac gene reactivation elicited by isoproterenol in NRVMs

**Figure 1:**
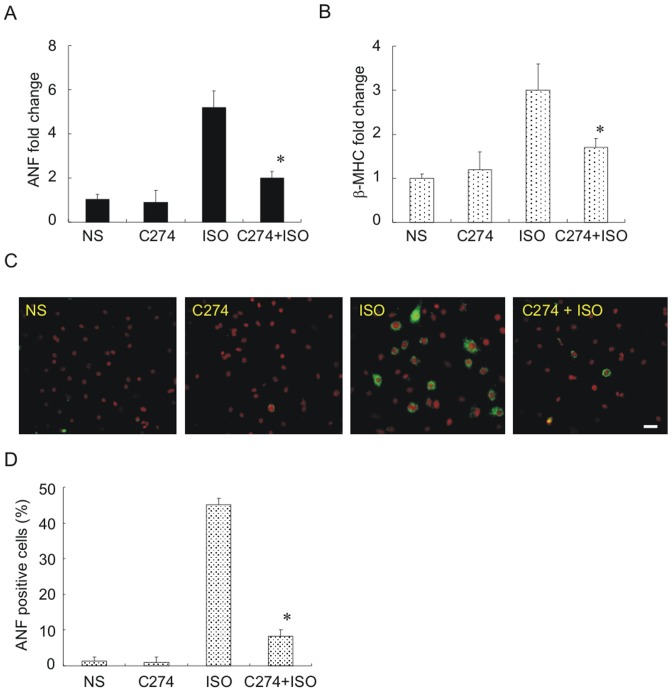
Isoproterenol-induced fetal gene upregulation is blunted by CpG-ODN in NRVMs. (A, B) induction of ANF (A) and β-MHC (B) mRNAs by isoproterenol as measured by qPCR assays and its blockade by pre-treatment with C274 (*n* = 4, * *p*<0.01 versus isoproterenol group). NRVMs were pre-treated with C274 (5 µg/ml) for 12 h followed by isoproterenol (10 µM×48 h). 18-S rRNA was used as an internal standard. The mean normalized value for expression of each gene in unstimulated cells is defined as 1. (C) the number of isoproterenol-induced ANF-expressing cells was reduced by C274 pre-incubation. For indirect immunofluorescence cells were first incubated with anti-ANF antibody followed by reaction with Alexa-488 conjugated second antibody (*green*). Propidium iodide (PI) was used as counterstain for nuclei (*red*). Scale bar = 20 µm. (D) quantitative analysis of ANF positive cells as shown in (C) (*n* = 4 throughout the groups, * *p*<0.01 versus isoproterenol group). For each experiment at least 50 cells were counted randomly using ImageJ.

Firstly, we evaluated the effect of C274, a C-type CpG-ODN, on pathological hypertrophy by employing a classic *in vitro* PCH model routinely used in our lab [6]. NRVMs were incubated with isoproterenol (10 µM x 48 h), and mRNA levels of the ANF and β-MHC were quantified using qPCR assays. As expected, isoproterenol treatment induced ANF and β-MHC expression by∼6-fold and 3-fold (Fig. 1A, B). However, pre-treatment of myocytes with C274 (5 µg/ml x 12 h) profoundly blunted isoproterenol's prohypertrophic effect. To determine whether the antihypertrophic effect is limited to C274 alone or a general function of all CpG-ODNs, we tested 1585 and 1826, representatives of A- and B-type CpG-ODNs, respectively. Results from these experiments indicated that 1585, but not 1826, exerted an antihypertrophic effect similar to C274 (Fig. S1). Thus, we conclude that both C- and A-type CpG-ODNs could antagonize isoproterenol-induced fetal gene re-expression.

We also monitored ANF expression at the protein level using the indirect immunofluorescent staining. Following isoproterenol treatment, prominent ANF signal was detected in peri-nuclear area of many cells (Fig. 1C) as we demonstrated recently [6]. Upon quantification, myocytes expressing ANF protein increased to∼50% following isoproterenol incubation. The percentage was significantly decreased by pre-incubation with C274 (Fig. 1D). C274 challenging alone had no effect on ANF-positive cell percentage (Fig. 1C, D). These results confirm that C274 stimulation could suppress isoproterenol-induced fetal gene re-activation.

**Figure 2:**
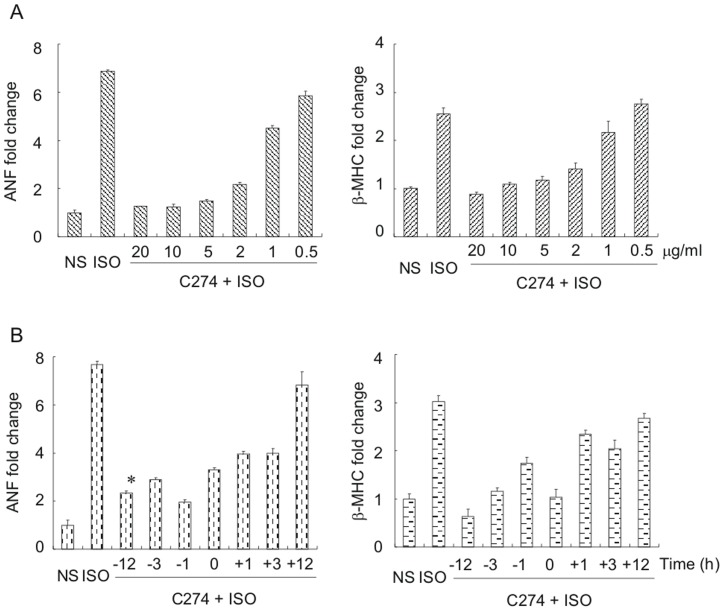
CpG-ODN represses isoproterenol-induced fetal gene reactivation in a dose- and time-dependent manner. (A) Effect of C274 (5 µg/ml) added at different time points on isoproterenol-induced gene reactivation. NRVMs were collected and assayed for ANF and β-MHC expression at different time points as indicated. (B) Dose-dependent effect of C274 on isoproterenol-induced gene reactivation. Myocytes were cultured with various doses of C274 for 12 h and then challenged with isoproterenol (10 µM x 48 h). Cells were collected and assayed for ANF and β-MHC expression. (# *p*<0.01, * *p*<0.05 versus isoproterenol group).

To determine the dose-dependent effect of C274, we pre-incubated NRVMs with different concentrations of the TLR9 agonist. The results showed that C274 at 1 µg/ml started to induce evident blockade of ANF and β-MHC expression provoked by isoproterenol (Fig. 2A). The anti-hypertrophic effect was increased profoundly when C274 concentration was raised from 1 to 20 µg/ml. In the kinetics assay, 5 µg/ml of C274 were added to NRVMs at several time points before or after isoproterenol incubation (10 µM x 48 h) (Fig. 2B). The results demonstrate that ANF and β-MHC expression evoked by isoproterenol was significantly suppressed by C274 added 12, 1 and 3 h before or 1, 3 h after isoproterenol treatment (*p*<0.05 versus ISO group). However, the protective effects decreased depend on the addition time of C274, and the effect was disappeared when C274 was added 12 h later (*p*>0.05 versus ISO group). These results suggest that C274 is most effective in preventing isoproterenol-induced hypertrophy but less effective when PCH is completed.

### 2. CpG-ODN prevents cell volume enlargement induced by isoproterenol in vitro

**Figure 3:**
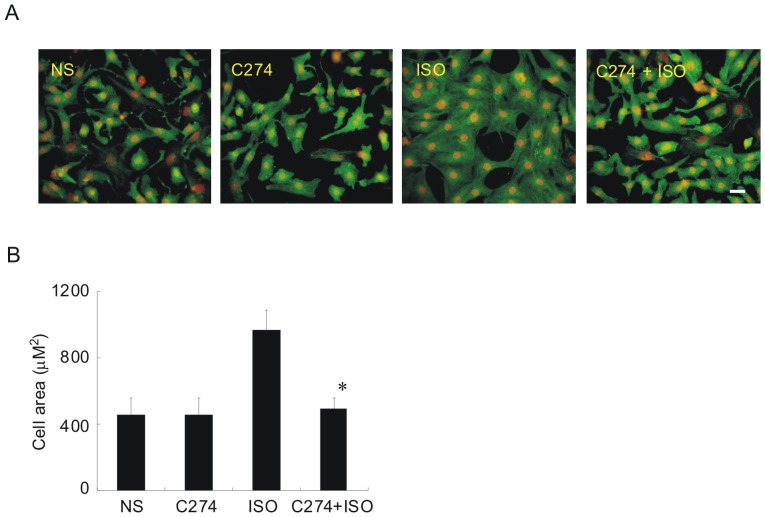
Isoproterenol-induced morphological alterations are prevented by CpG-ODN pre-incubation. (A) immunolocalization of α-actinin in NRVMs. Cells were incubated with an anti-α-actinin followed by reaction with Alexa-488 conjugated second antibody (*green*). PI was used as counterstain for nuclei (*red*). Cells were pre-treated with C274 (5 µg/ml x 12 h) followed by isoproterenol (10 µM x 48 h). Note the highly-organized sarcomeres in isoproterenol-treated cells. Scale bar = 20 µm. (B) quantitative analysis of cross-sectional area for each group as shown in (A) (*n* = 3, *p<0.05 vs isoproterenol group). For each experiment at least 50 cells were estimated randomly using ImageJ.

We next examined how other pathological hypertrophic markers would be affected by the TLR9 agonists. To this end, cell size and sarcomere organization were measured by staining myocytes for α-actinin. Remaining in culture for 48 h, cells treated with normal saline (NS) appeared more round-shaped with soft edges and disorganized sarcomeres (Fig. 3A). The average cross-sectional area, which is used as a surrogate of cell volume, was∼400 µm^2^ for control myocytes (Fig. 3B) while stimulation with isoproterenol for 48 h resulted in the appearance of highly-organized sarcomeres with sharp edges and increased cell area (Fig. 3). Typically, isoproterenol-treated cells increased their cross-sectional area to∼1000 µm^2^ under our experimental conditions [6]. However, when the cell was pre-incubated with C274, the cell size had no appreciable change after isoproterenol stimulation (Fig. 3). CpG-ODN alone had no effect on the cell size. These morphological results further support the idea that CpG-ODN could prevent the NRVM from developing PCH under isoproterenol challenging.

### 3. PI3Kα-Akt pathway is essential for the antihypertrophic effect of CpG-ODN in NRVMs

**Figure 4:**
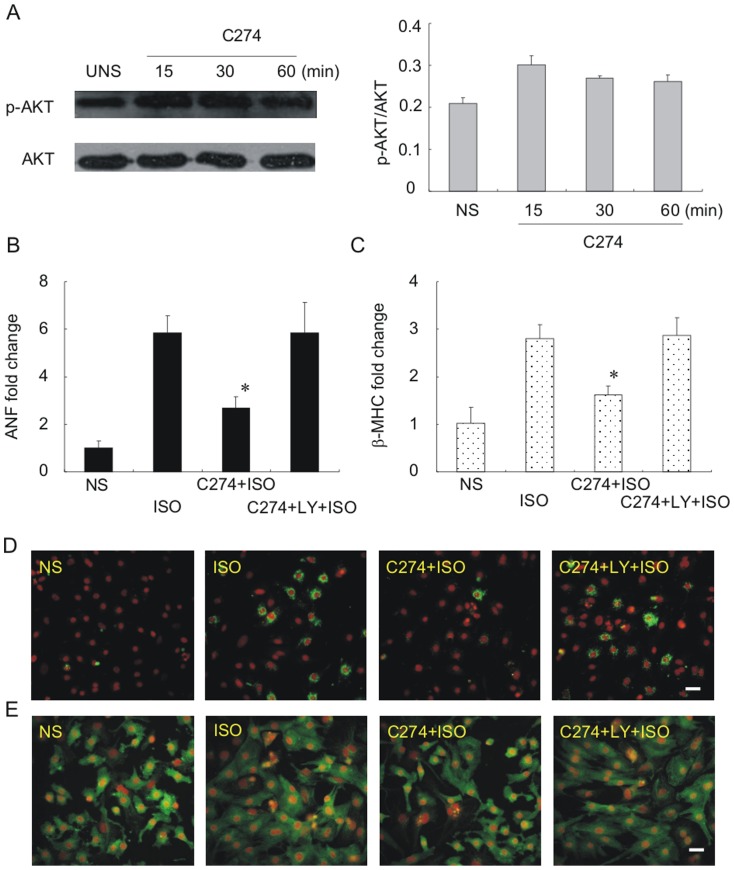
PI3K-Akt signaling pathway is required for the antihypertrophic effect of CpG-ODN. (A) induction of Akt phosphorylation by C274 (5 µg/ml x 12 h) as demonstrated by Western blot assays. Plotted on the right is quantitative analysis of data shown in the left panel. Band density was determined and analyzed using ImageJ. (B, C) isoproterenol-induced increase in ANF and β-MHC mRNAs (*second bars from left*) was inhibited by C274 (*third bars from left*) but no longer inhibited by C274 in cells pre-incubated with a pan-PI3K inhibitor LY294002 (*fourth bars from left*). (D) C274 failed to prevent isoproterenol-induced ANF expression at protein level in cells pretreated with LY. (E) C274 failed to normalize isoproterenol-induced morphological alterations in myocytes pretreated with LY. Scale bar = 20 µm.

Increasing evidences show that activity of PI3K was required for TLR9-mediated induction of type I interferon in response to CpG-ODNs [22]. Although PI3Kγ-activated Akt signaling is prohypertrophic, PI3Kα-Akt pathway is an important mediator of physiologic growth. We thus decided to measure phosphorylated Akt (p-Akt), a downstream target of PI3Ks. Interestingly, we found constitutive Akt phosphorylation at position Ser473 in the NRVM even in the absence of CpG-ODN stimulation. However, p-Akt was further significantly up-regulated in NRVMs after 15, 30 and 60 min incubation with C274 (Fig. 4A).

We then determined the functional consequences of disrupting the PI3K-Akt signaling pathway in pathological hypertrophy induction. LY294002, a pan-PI3K inhibitor, was used to suppress PI3Ks. Consistent with the results shown in Fig. 1, isoproterenol readily up-regulated the expression of ANF (Fig. 4B) and β-MHC (Fig. 4C), and pre-incubation with C274 markedly antagonized the isoproterenol-induced fetal gene expression (Fig. 4B, C). However, isoproterenol-induced increase in the ANF and β-MHC mRNAs was no longer inhibited by C274 in cells pre-incubated with LY294002 (Fig. 4B, C). C274 also failed to prevent isoproterenol-induced ANF expression at the protein level (Fig. 4D) as well as the morphological alterations (Fig. 4E) in myocytes pretreated with LY294002. These results demonstrate that inhibition of PI3Ks with LY294002 could suppress CpG-ODN-induced antihypertrophic effect.

**Figure 5:**
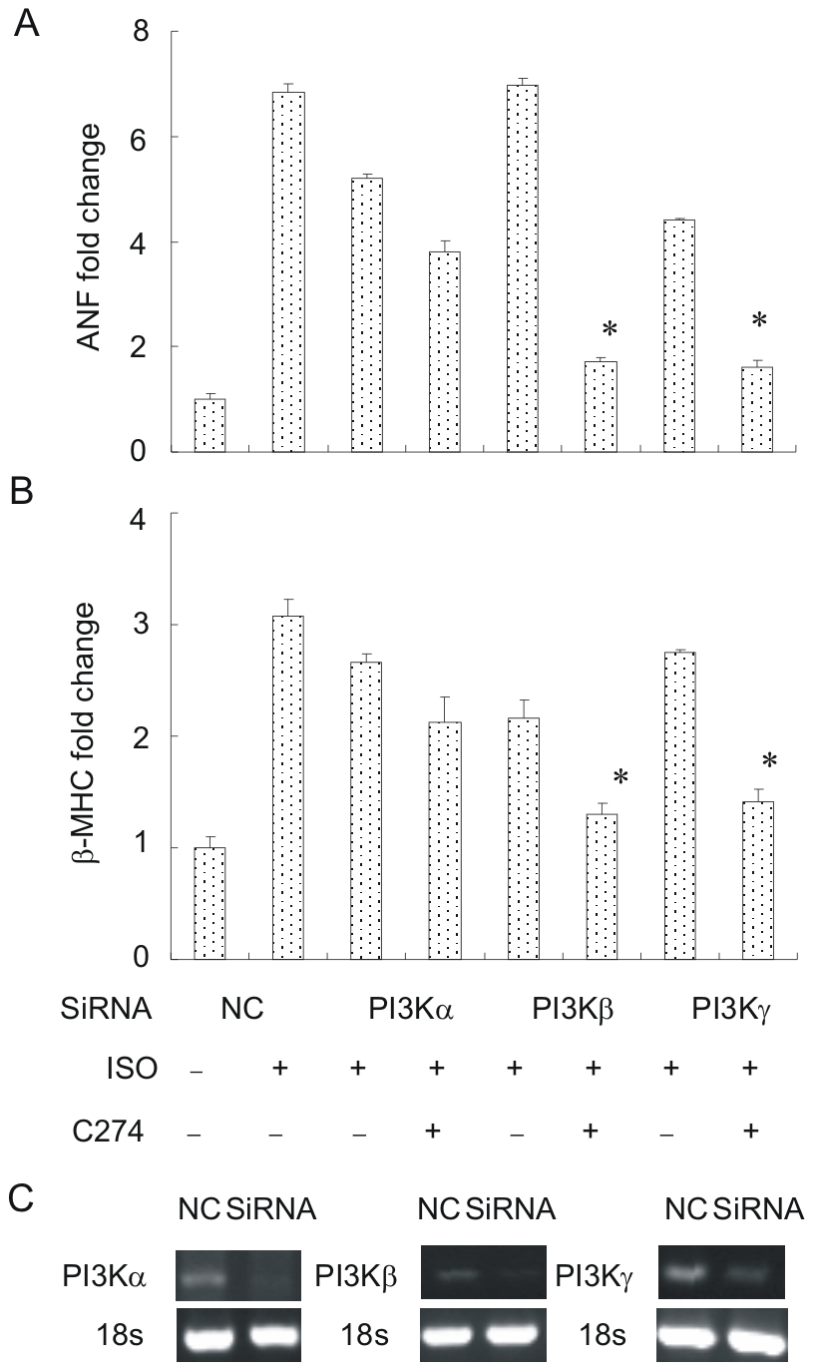
Gene silencing of PI3Kα cancels out CpG-ODN's antihypertrophic effect in NRVMs. (A, B) Effects of gene silencing of different PI3K isoforms on C274's antihypertrophic efficacy marked by ANF (A) and β-MHC mRNAs (B). Isoproterenol was used to induce PCH, and ANF and β-MHC mRNAs were used as PCH markers quantified by qPCR assays. C274 pretreatment significantly blunted isoproterenol-induced expression of ANF (A) and β-MHC (B) but failed to do so in cells with gene knock-down of PI3Kα (A, B). C274 pretreatment still significantly prevented isoproterenol-induced expression of ANF (A) and β-MHC (B) in cells with gene knockdown of PI3Kβ or PI3Kγ (A, B). Neonatal myocytes were transfected for 6 h with control or siRNAs specific to PI3Kα, PI3Kβ or PI3Kγ. 36 h later, cells were incubated with C274 (5 µg/ml×12 h) followed by isoproterenol (10 µM×48 h). (*p<0.05 versus isoproterenol group). (C) RT-PCR assays showing gene silencing efficacy for PI3Kα, PI3Kβ and PI3Kγ. 18-S rRNA serves as a loading control.

Specificity of signaling inhibitors such as LY294002 could be an issue, especially when they remain in cultures exceeding several hours. In order to exclude any off-target effect of LY294002, small interfering RNA (siRNA) was used to specifically knock down PI3Kα, PI3Kβ and PI3Kγ. mRNA levels of the ANF (Fig. 5A) and β-MHC (Fig. 5B) were used as hypertrophic markers and gene silencing efficacy was verified by RT-PCR (Fig. 5C). As predicted, silencing of PI3Kα largely prevented the C274's antihypertrophic effect as the expression levels of both fetal cardiac genes was elevated by isoproterenol, whereas silencing of PI3Kβ or PI3Kγ showed no appreciable effect (Fig. 5A, B). These results confirm that PI3Kα is crucial for CpG-ODN's antihypertrophic effect in the NRVM.

### 4. CpG-ODN could suppress cardiac dysfunction and PCH induced by isoproterenol in vivo

**Figure 6:**
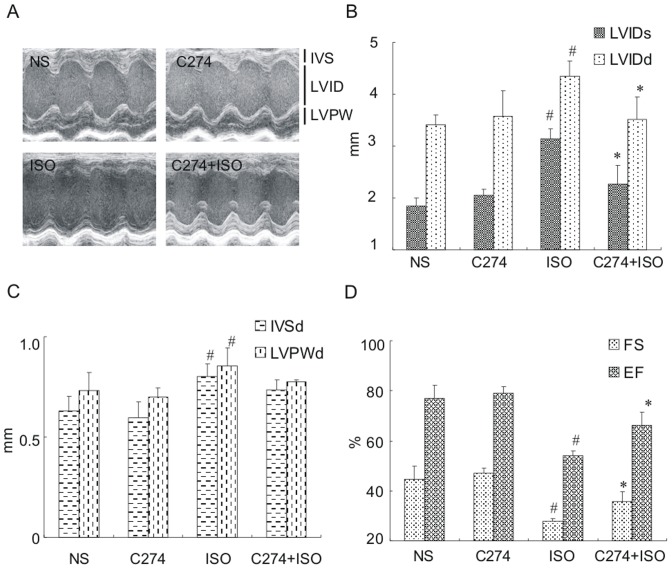
C274 attenuates echocardiographic changes induced by isoproterenol ***in vivo*** **.** (A) Shown are representative long-axis parasternal echocardiographic images. IVS, interventricular septum; LVPW, left ventricular posterior wall; LVID, left ventricular internal dimension. Mice (6–8 in each group) were subjected to treatment with vehicle control (NS), C274 (5 mg/kg, i.p.), isoproterenol (50 mg/kg, s.c.) or isoproterenol preceded by C274 (5 mg/kg, i.p.) for 6 d. Echocardiography was performed under inhalational isoflurane/oxygen anasthesia 24 h after last drug injection. (B, C) Comparison of IVSd, LVPWd and LVIDs, LVIDd for animals shown in (A) (*n* = 6-8, # *p*<0.05 versus NS group, * *p*<0.05 versus ISO group, Student's *t* test). (D) Comparsion of fractional shortening (FS) and ejection fraction (EF) for animals shown in (A) (*n* = 6-8, # *p*<0.05 versus NS group, * *p*<0.05 versus ISO group, Student's *t* test). FS  =  (LVIDd-LVIDs)/LVIDd×100 and EF  =  (LVIDd^3^- LVIDs^3^)/LVIDd^3^×100.

To determine whether CpG-ODN could inhibit pathological hypertrophy and cardiac dysfunction *in vivo*, we performed echocardiographic analysis in mice with various drug treatments. The first group of C57BL/6 mice was injected with C274 (5 mg/kg) intraperitoneally (i.p.), followed by subcutaneous (s.c.) injection of isoproterenol (50 mg/kg) 3 h later. This injection scheme was repeated for 6 d. Application of isoproterenol, C274 or NS alone was also performed in additional animals and mice were subjected to cardiac echo 24 h after the last drug application. Representative cardiac echo images and measured parameters are depictured in Fig. 6. As expected, mice with isoproterenol injection showed an enlarged left ventricular chamber size as reflected by a significant increase in left ventricular internal dimension values at end diastole (LVIDd) and end systole (LVIDs), compared to the NS control (Fig. 6B). Isoproterenol also resulted in significant wall thicking as evidenced by increased values in interventricular septal (IVSs) dimensions at end systole, and left ventricular post wall thickness at end diastole (LVPWd) (Fig. 6C). Consistently, these structural alterations were accompanied by marked impairment in cardiac contractile function as represented by a decrease in left ventricular fractional shortening (FS), and ejection fraction (EF) (Fig. 6D). By comparison, pretreatment with C274 greatly reversed changes in almost all above parameters evoked by isoproterenol (Fig. 6).

**Figure 7:**
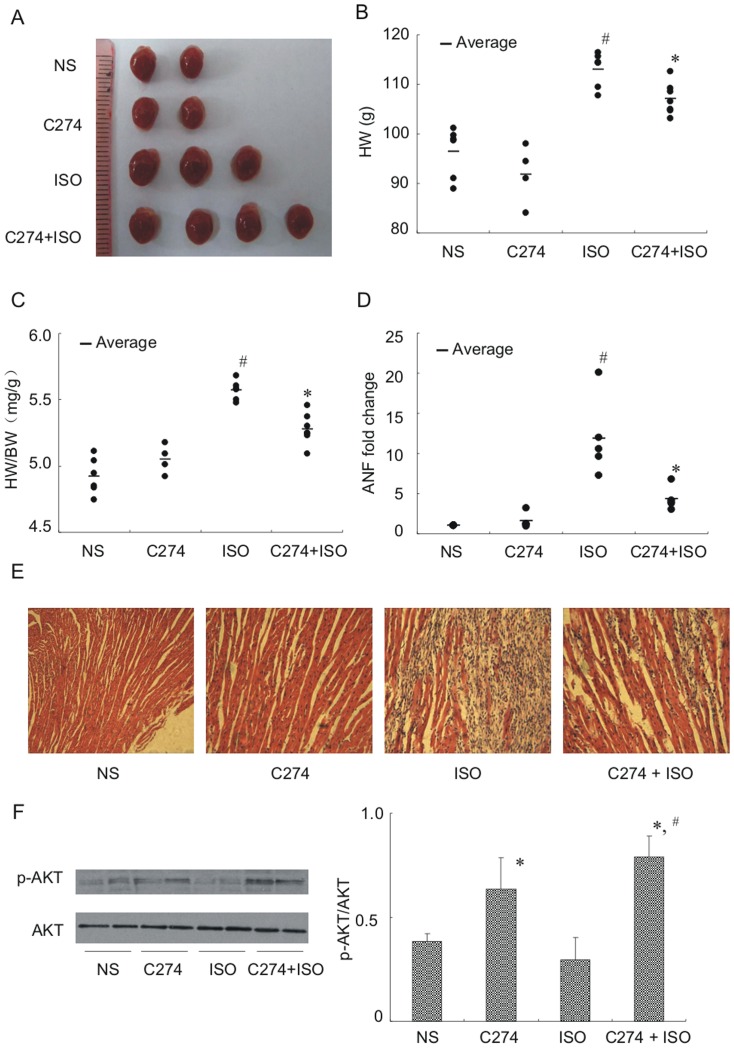
C274 prevents isoproterenol-induced cardiac remodeling *in vivo*. (A) Representative whole heart pictures taken from mice with treatments as indicated. Mice (6-8 in each group) were subjected to injection with vehicle control (NS), C274 (5 mg/kg, i.p.), isoproterenol (50 mg/kg, s.c.) or isoproterenol preceded by C274 (5 mg/kg, i.p.) for 6 d. 24 h after last drug injection, animals were sacrificed and hearts were removed for hypertrophic evaluation. (B, C) Comparison of HW (g) and HW/BW ratios (mg/g) for animals shown in (A) (*n* = 6-8, # *p*<0.05 versus NS group, * *p*<0.05 versus ISO group, Student's *t* test). (D) Comparison of ANF mRNA in the hearts of animals as shown in (A) (*n* = 6-8, # *p*<0.05 versus NS group, * *p*<0.05 versus isoproterenol group, Student's *t* test). (E) Histological sections of hearts by H&E staining. Massive cardiomyocyte death is demonstrated by missing myocytes replaced by fibrosis intermingled with inflammatory cells in the isoproterenol-treated heart. The sections were photographed under 60×microscopy. (F) Representative Western blotting assays and quantitative analysis of phosphorylated Akt in the hearts with indicated drug treatments (* *p*<0.05, versus isoproterenol group, # *p* = 0.14 vs C274, Student's *t* test).

To further verify CpG-ODN's antihypertrophic effect, we directly measured heart weight (HW) and heart to body weight (HW/BW) ratio by sacrifying the animals. We also analyzed histological sections by hematoxylin & eosin staining and quantitated ANF mRNA by qPCR assays. As expected, isoproterenol caused a significant increase in HW (Fig. 7A, B), HW/BW ratio (Fig. 7C) and ANF expression (Fig. 7D), compared with the NS control. Because cardiac function was markedly impaired by isoproterenol as demonstrated by echocardiography (Fig. 6), cardiac cell death was expected. Consistently, massive cell death with replacement fibrosis was observed in the isoproterenol-treated hearts (Fig. 7E). By comparison, C274-pretreatment abbreviated isoproterenol-elicited alterations in all above hypertrophic markers (Fig. 7A-D) and cardiac cell death/fibrosis (Fig. 7E). Western blot analysis revealed significant enhanced Akt phosphorylation in the C274-treated hearts (Fig. 7F) despite total Akt protein being similar in all animal groups. The C274-isoproterenol double-injection animals seemed to have higher p-Akt in the hearts than that treated with C274 alone, but the difference did not reach a significant level (*n* = 4, # *p* = 0.14 vs C274 group, Student's *t* test). These results indicate that C274's cardioprotective effect could be attributed to both of the antihypertrophic and anti-cell death activity, and the PI3Kα-Akt signaling pathway is likely involved in these beneficial responses.

## Discussion

Our results can be summarized as follows: (1) Both C-type and A-type CpG-ODNs could antagonize hypertrophy induced by isoproterenol *in vitro*; (2) The antihypertrophic effect of CpG-ODNs requires PI3Kα activity, which is associated with enhanced Akt phosphorylation; (3) Pre-treatment with a CpG-ODN C274 could suppress PCH induced by isoproterenol *in vivo*; and (4) C274 also abbreviates cardiac cell death, replacement fibrosis, and resultant cardiac dysfunction induced by isoproterenol *in vivo*. These results corroborate the existing idea that PCH and cardiac dysfunction can be antagonized by boosting the PI3Kα-Akt physiologic growth pathway [3], [9].

One way of activating the PI3Kα-Akt signaling is by physical exercise, but this is impractical in patients of cardiac dysfunction. Thus, it is logical to search for exercise mimetic to serve the same purpose. Here we have demonstrated that CpG-ODNs are activators of the PI3Kα-Akt signaling in cardiomyocytes. Results similar to ours have also been reported recently by independent labs, although in their case, cardiac dysfunction was induced by reperfusion injury, hemorrhagic shock, or sepsis [23], [24]. In addition, cardioprotection has been observed for TLR2 agonists in a PI3K-Akt dependent fashion [25]. Although current data point to an important role played by the PI3Kα-Akt signaling in the cardioprotection, elucidation of the exact mechanisms requires future work to be performed in mice lacking Akt or PI3Kα.

Cardiac cell death is another prerequisite for heart failure to occur. Apparently, one of the mechanisms underlying CpG-ODN's cardioprotection lies in the prevention of cardiac cell death as reflected by less fibrosis, although we did not address this issue in full detail. Previous studies have demonstrated that mice overexpressing PI3K-p110α or Akt are protected from heart failure via inhibition of cell death pathways [26]. Chronic exercise training reduces infarct size and inhibits cardiac cell death in rats subjected to reperfusion injury while treatment with a PI3K inhibitor prior to reperfusion reverses the beneficial effects of exercise training [27]. Thus, the cardioprotective effect of CpG-ODNs is partially attributed to the activation of anti-cell death pathways involving PI3Kα-Akt signaling.

In addition to TLR9, other TLR members including TLR2, 3, 4, 5, and 7 are identified in cardiomyocytes, and are also verified in the NRVM and H9c2 cells in the present study. Although PI3K/Akt is a signaling pathway common to all the TLRs, only activation of TLR9 (this study) and TLR2 [25] is cardioprotective. In contrast, activation of TLR4 results in a robust inflammatory response and decreases cardiac contractility [17]. Gene silencing of NFκB, a downstream target for TLR4, prevents PCH and its progression to heart failure in a transgenic myotrophin model [28] while TLR4-deficient mice are resistant to pressure overload-induced PCH and heart failure [29]. What makes the difference is presently unknown, but it is probable that systematic inflammation caused by TLR4 activation overwhelms its potential cardioprotective effect. Further investigation is required to dissect the underlying distinct mechanisms.

A limitation of this study is that only an isoproterenol-induced hypertrophic model was used for the *in vivo* test of CpG-ODN's antihypertrophic effect. The isoproterenol model is well-established in our laboratory [6] and convenient to generate and use. The model animals manifest prominent pathological hypertrophy, increased ANF expression, and massive cell death with replacement fibrosis, recapitulating the major aspects of PCH and heart failure in human subjects [30], [31]. Our results obtained from this model also showed consistent and reproducible morphological and echocardiographic alterations characterizing PCH and heart failure, which justify the suitability of the model for our purpose. It would be desirable to use additional PCH models to test our results such as thoracic aortic constriction (TAC), which is widely accepted and of clinical relevance. This will be performed in the future studies.

## Supporting Information

Figure S1 **Isoproterenol-induced fetal gene expression was blunted by CpG ODN 1585, C274 but not CpG ODN 1826 or other TLR agonists in neonatal myocytes.** (A, B) induction of ANF (A) and β-MHC (B) mRNAs by isoproterenol as measured by qPCR assays and its blockade by pre-treatment with CpG ODN 1585 or C274. Neonatal myocytes were pre-treated with PGN (10 µg/ml, TLR2 agonist), PolyI:C(25 µg/ml, TLR3 agonist), LPS (1 µg/ml, TLR4 agonist) or CpG ODN 1585, 1826 or C274 (5 µg/ml, TLR9 agonist) for 12 h followed by ISO stimulation for 48 h. 18-S rRNA was used as an internal standard. The mean normalized value for expression of each gene in unstimulated cells is defined as 1.
(DOC)
