# Supplementary figures and images for: CpG-ODN Attenuates Pathological Cardiac Hypertrophy and Heart Failure by Activation of PI3Kα-Akt Signaling
Source: PLoS One. 2013 Apr 30;8(4):e62373. doi: 10.1371/journal.pone.0062373 (PMC3640052; doi:10.1371/journal.pone.0062373)

**Figure S1**


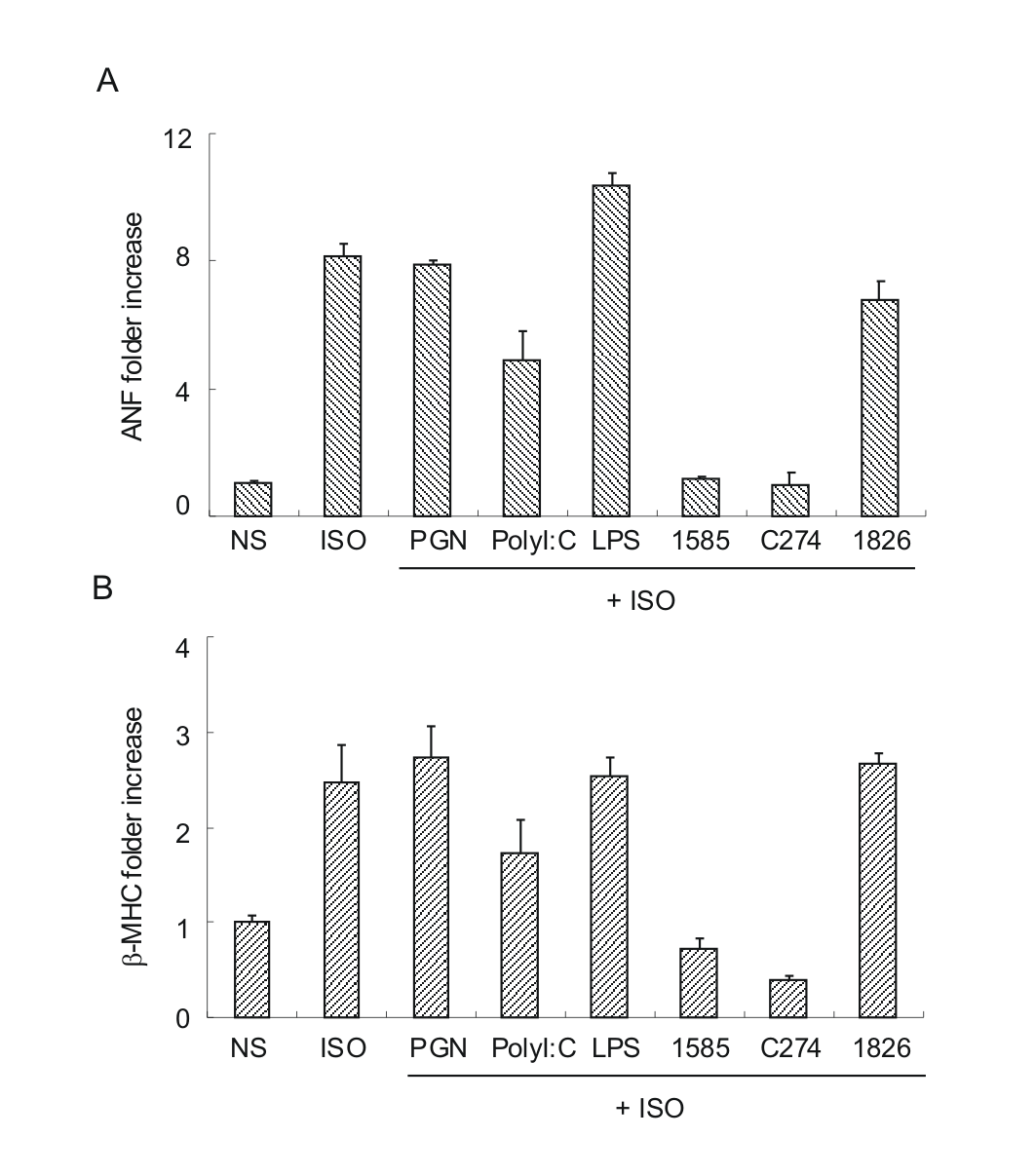

Supplement: Figure S1 — Isoproterenol-induced fetal gene expression was blunted by CpG ODN 1585, C274 but not CpG ODN 1826 or other TLR agonists in neonatal myocytes. (A, B) induction of ANF (A) and β-MHC (B) mRNAs by isoproterenol as measured by qPCR assays and its blockade by pre-treatment with CpG ODN 1585 or C274. Neonatal myocytes were pre-treated with PGN (10 µg/ml, TLR2 agonist), PolyI:C(25 µg/ml, TLR3 agonist), LPS (1 µg/ml, TLR4 agonist) or CpG ODN 1585, 1826 or C274 (5 µg/ml, TLR9 agonist) for 12 h followed by ISO stimulation for 48 h. 18-S rRNA was used as an internal standard. The mean normalized value for expression of each gene in unstimulated cells is defined as 1. (DOC) [file pone.0062373.s001.doc]
